# Letter from Dr. Welch

**Published:** 1848-02

**Authors:** W. W. Welch

**Affiliations:** Chicago, Ill.


ARTICLE III.

We take the liberty of publishing the following extract from a private letter, dated Inlet Grove, Lee county, January 20, from our esteemed young friend Dr. Welch.—Ed.

"I have had a *curious*, and, perhaps, dangerous case, upon which I should like your opinion. In the month of October, I was sent for to see a little girl, of seven years of age, who was said to be barking like a dog. I found her to be, to all appearance, in health, except that she had, every few minutes, a paroxysm of a peculiar ringing spasmodic cough, very difficult to describe, but as near as anything not unlike the rapid barking of a small dog. There was no pain, nor difficulty of breathing, nor expectoration, nor sense of oppression during or between the paroxysms; which occurred, perhaps, four or five times in the course of an hour, lasting not above a minute or two. An emetic relieved her for the time and thinking *malaria* might have something to do with it, gave quinine to an extent to produce nausea, whenever they should occur again. She gradually, for a time, got better under this treatment, but at length, in six or eight weeks they began to recur again with more severity, and my assistance was once more required. The cough had changed, somewhat, its character, being less ringing; and sometimes there would be scarcely any noise, but the mere pantomine of a cough. There was some oppression now during the cough, and sometimes she would have frequent vomiting without nausea. Blistered the back of the neck; gave assafœtida with camphor and ipecac and more quinine. Improved under this treatment for a time; but two weeks since became worse, and being sent for again, I examined the tonsils which were found to be much enlarged, injected and studded with a few white points. They seemed to be, besides, quite sensitive, a touch being enough to excite the spasmodic cough. I thought this enlargement might have something to do in producing the difficulty, and proposed that the glands should be removed, but as the parents would not consent, I substituted astringent gargles, and resorted again to assafœtida. The cough gradually left her under this plan, in the course of a week; but this being subdued, she began to have involuntary contractions of the muscles of the back, resembling St. Vitus' dance, and continuing about the same length of time with the previous paroxysms of coughing; the other muscles of the body not seeming to participate in the convulsive movements. The child now ejected almost everything that she took into the stomach; but there was no nausea or unpleasant sensation at the time. She was, also, becoming considerably emaciated; I put her upon acourse of carb. of iron, in doses of, perhaps, four to five grains, with assafœtida, when the paroxysms were most frequent. And as her meals were thrown up, I directed food to be given in small quantities at a time, often, and no more than could be easily retained. Under this course she has rapidly improved; the paroxysms being much less severe, and less frequent, her strength is improving and she no longer ejects her food, when given as directed.

I should value highly any remarks you may be pleased to make upon the above case. I should have mentioned, that the child has a light complexion, and is fair, and has always been well, save that she has sometimes, not very often, thrown up her food. The parents too, though not remarkably vigorous, cannot be considered unhealthy.

I will crave but another minute of your time, just to tell you how the women increase and multiply the population here. Last fall, a woman living in this vicinity, brought forth three living children at one birth; one died after a time, of disease, the other two are still living.

Day before yesterday, (20th,) I had just left the house where I had attended a case of childbirth, when I was called in another direction. The woman was but seven months advanced, and my advice was sought rather on the account of extreme anasarca of the legs and thighs, *and great* œdema of the labia pud., than in expectation of my services being needed obstetrically. She, however, was begining to have premonitory symptoms of approaching labor, and I disposed myself accordingly; thinking the labia might suffer injury from the passing of the child, I made a number of small punctures with a lancet in each, by which the swelling was reduced, and a large quantity of serum evacuated during the course of the labor. The abdomen was enormously enlarged, and the labor was slow, lasting some twenty-four hours. It had been apparently near its termination for several hours, when two fœtuses, in appearance of about six months growth, were suddenly expelled. One was in a state of incipient putrefaction, and the other, barely showed signs of life. Soon after, in searching for the after-birth, my fingers met a very large watery cyst, which as contractions proceeded, was soon ruptured, and an apparently seven months fœtus was delivered by the feet. It was inanimate and there was no pulsation in the cord; but artificial respiration being instituted, it was after a time resuscitated.

Making still another examination after the lapse of a few minutes, the head of a *fourth* child was found to be rapidly advancing, and it was soon in this vale of tears. (I had to laugh when the woman asked me if I thought that was the last one.) It was more vigorous than the other, and the cord pulsated strongly. The two double placentæ were then extricated; the last adhering, slightly, it being necessary to detatch it carefully with the fingers.

There was some hemorrhage but it was speedily arrested, and the woman was "comfortable and happy" when I left her yesterday.

The two first born were girls, the two last boys.

Respectfully, yours,

W. W. WELCH.

To Dr. Brainard,
Chicago, Ill.

